# Seasonal soil health dynamics in soy-wheat relay intercropping

**DOI:** 10.1038/s41598-024-69903-5

**Published:** 2024-08-16

**Authors:** Jennifer B. Thompson, Thomas F. Döring, Timothy M. Bowles, Steffen Kolb, Sonoko D. Bellingrath-Kimura, Moritz Reckling

**Affiliations:** 1 Leibniz Centre for Agricultural Landscape Research (ZALF), 15374 Müncheberg, Germany; 2 Faculty of Life Science, Thaer-Institute of Agricultural and Horticultural Science, Humboldt-University of Berlin, 14195 Berlin, Germany; 3 Institute of Crop Science and Resource Conservation, Agroecology and Organic Farming, University of Bonn, 53121 Bonn, Germany; 4 Department of Environmental Science, Policy and Management, University of California, Berkeley, CA USA; 5 Department of Crop Production Ecology, Swedish University of Agricultural Sciences (SLU), Uppsala, Sweden

**Keywords:** Spatial diversification, Crop mixture, Soil carbon, Soil microbial communities, Environmental sciences, Agroecology

## Abstract

There is growing interest in intercropping as a practice to increase productivity per unit area and ecosystem functioning in agricultural systems. Relay intercropping with soy and winter wheat may benefit soil health due to increased diversity and longer undisturbed soil cover, yet this remains largely unstudied. Using a field experiment in Eastern Germany, we studied the temporal dynamics of chemical, biological, and physical indicators of soil health in the topsoil over a year of cultivation to detect early effects of soy-wheat relay intercropping compared to sole cropping. Indicators included microbial abundance, permanganate-oxidizable carbon, carbon fractions, pH, and water infiltration. Relay intercropping showed no unique soil health benefits compared to sole cropping, likely affected by drought that stressed intercropped soy. Relay intercropping did, however, maintain several properties of both sole crops including an increased MAOM C:N ratio and higher soil water infiltration. The MAOM C:N ratio increased by 4.2 and 6.2% in intercropping and sole soy and decreased by 5% in sole wheat. Average near-saturated soil water infiltration rates were 12.6, 14.9, and 6.0 cm hr^−1^ for intercropping, sole wheat, and sole soy, respectively. Cropping system did not consistently affect other indicators but we found temporal patterns of these indicators, showing their sensitivity to external changes.

## Introduction

There is growing interest in diversified farming systems as a means to simultaneously enhance ecosystem services and productivity per unit area. Conventional agricultural practices have long been associated with a multitude of environmental challenges including soil erosion, depletion of soil carbon, and greenhouse gas emissions^1–3^. Therefore, it is crucial to identify agricultural management that ensures long-term productivity and stability while minimizing adverse environmental impacts. Diversified farming systems generally include crop diversification which can be achieved temporally with crop rotation and spatially with intercropping^4^, where multiple crops are cultivated together on a single field. Intercropping offers farmers numerous options for spatial arrangements, promoting diversity by incorporating a greater variety of crop types, varieties, and functional groups into their operation^5^.

Besides showing potential for higher productivity than sole crops^6–9^, intercropping may be a useful agricultural management practice to support soil health. Soil health, defined by the US Department of Agriculture^10^ as “the continued capacity of soil to function as a vital living ecosystem that sustains plants, animals, and humans”, supports multiple ecosystem services beyond crop production, including nutrient and water cycling. While the term soil health has been defined in many ways^11–13^, it is an increasingly common way of studying and managing soils and is now a priority in the EU Soils strategy of 2030. Soil health is commonly measured with a suite of chemical, physical, and biological indicators including, but not limited to, soil organic carbon (SOC), nitrogen pools, soil aggregation, and soil compaction. Intercropping wheat, maize, and legumes can increase soil organic carbon and nitrogen^14–16^ which has been attributed to the increased root biomass input in intercropping^14^. Intercropping also enhances root exudate diversity and abundance which can stimulate microbial activity and abundance^17–19^. Similarly, intercropping has also been shown to increase soil aggregation^15^, decrease bulk density^20^, and increase microbial biomass and soil enzyme activity^21–23^, all of which contribute to soil health.

Relay intercropping involves seeding a second crop in between an already established crop prior to its harvest, leading to two simultaneously growing crops with different harvest times. It offers security over double cropping in regions where the growing season may be too short for the second crop to mature^24^. Relay intercropping has been less researched than other types of mixed cropping^25^ and adoption is not widespread—primarily restricted to China, North America, and regions in Africa. Relay cropping can be productive and profitable with some studies finding benefits for disease and pest reduction^26,27^. Relay intercropping, with its distinct field arrangement, also holds promise for promoting soil health. Like mixed cropping, relay intercropping can lead to increased soil nutrient utilization from the two different crops using different resource niches^26^, producing diverse root exudates^17,19^, and increasing root biomass^14^ all of which have positive implications for soil health. Due to relay intercropping’s longer season, relay intercropped fields will have one crop still growing when sole cropped fields have already been harvested and left fallow in regions where only one crop per season is possible. The extended duration of relay intercropping compared to single-cropped fields allows for the maintenance of living roots, minimized tillage compared to double crops, increased soil cover, reduced soil erosion, and crop biodiversity, which align with the USDA's principles of maintaining soil health.

Relay intercropping with soy and wheat presents an opportunity for farmers to enhance the diversity of their cropping systems. The system offers crops that have been extensively proven to be profitable (wheat) and those that are still relatively novel in central Europe but have high demand (i.e. soybean^28^). In Western and Central Europe, where rotations are dominated by cereals and oilseeds^29^, intercropping a legume could be a strategy to increase legume adoption into rotations as cereal and legume intercropping is an already established combination with good resource complementarity. Intercropping systems are context-specific, with results contingent upon factors such as region and crop combination; yet, no information is available regarding the impact of soy-wheat relay intercropping on soil health, although literature on relay intercropping soy with other cereals exists^30^. Moreover, there is a further knowledge gap regarding how quickly relay intercropping could lead to measurable changes in soil health—whether it could provide short-term benefits after a single season of cultivation or whether farmers would need to cultivate it for many years before seeing benefits. Consequently, the effectiveness of this diversification strategy in promoting soil health remains largely unknown.

Assessing soil health in a relay intercropping system poses challenges as it involves two crops that overlap for only a portion of the growing season. Determining how and when to measure indicators is complex and relying on single sampling (even if replicated over years or sites) may inadequately evaluate the system or hinder understanding of a specific crop's influence the soil. Thus, sensitivity of soil health indicators is particularly important in relay intercropping. Traditionally, assessments of soil health rely on a range of indicators, some of which, like total SOC can take years or decades to exhibit significant changes^13,31^. While undeniably valuable, such methods may be better suited for long-term experiments and less feasible for newer agricultural practices which may undergo trials lasting only one or two seasons. To address this limitation, we propose the incorporation of more rapidly responsive soil health indicators to monitor changes.

The primary objective of this case study was to identify sensitive soil health indicators and short-term effects associated with soy-winter wheat relay intercropping compared to sole cropping throughout the complete life cycle of both crops. Utilizing a field experiment, our study included an assessment of biological, chemical, and physical indicators of soil health five times over an entire year. Indicators were selected to be sensitive to management and informative to soil ecosystem services of interest. By adopting this approach, we aimed to develop a comprehensive understanding of suitable indicators and the dynamics governing soil health in the context of soy-wheat relay intercropping. We hypothesized that the enhanced spatial and temporal diversification in relay-intercropping would improve soil health indicators by increasing bacterial and fungal functional diversity and abundance. Moreover, we hypothesized that relay intercropping would lead to small but measurable increases in soil C fractions, POXC, and water infiltration rates.

## Results

### Soil health chemical indicators

**Figure 1:**
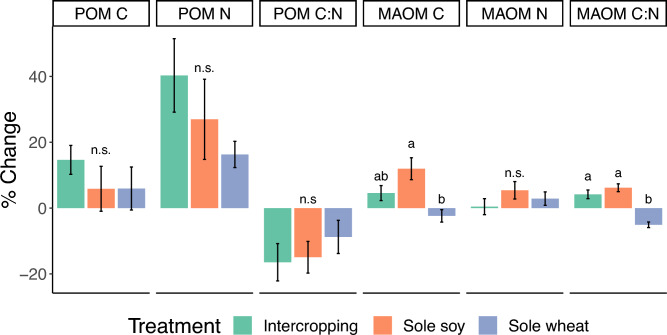
Percent change in carbon fraction values between the start to the end of the cropping season. Bars depict standard error and letters show significance.

The mineral-associated organic matter (MAOM) C:N percent change (i.e. the change from the start to the end of the cropping season, hereafter called ∆) was higher in intercropping (Fig. 1, p < 0.001) and sole soy (p < 0.001) than sole wheat but there was no difference between intercropping and sole soy (p = 0.42). There was no difference in ∆MAOM C between sole soy and intercropping (p = 0.06) but sole wheat’s overall percent decrease in ∆MAOM C was significantly lower than sole soy (p < 0.0001). Final MAOM %C values were 2.02%, 1.85%, and 1.72% for sole soy, intercropping, and sole wheat, respectively (Supplementary Table 2). There was no significant difference in ∆MAOM N between cropping systems. As for particulate organic matter (POM), we found no significant differences in ∆POM C, ∆N or ∆POM C:N but there was an overall increase of both ∆POM C and ∆POM N for all treatments.

**Figure 2:**
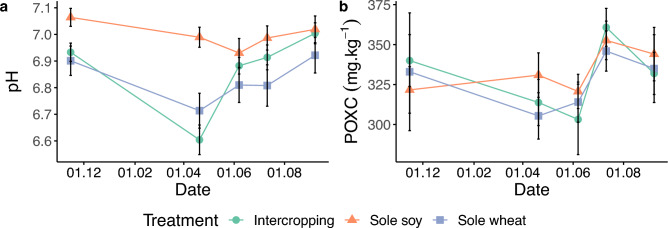
(**a**) Soil pH and (**b**) POXC over a cropping season. Bars show standard error.

There was a significant effect of sampling time (*p* < 0.001, Fig. 2), treatment (p = 0.004), and a treatment*sampling interaction (p = 0.0004) on soil pH. The average soil pH decreased to its lowest point of 6.77 at Sampling 2 (at the time of wheat fertilization) where it was lower than every other sampling (p < 0.001 for all pairwise comparisons) but increased again until Sampling 5. pH was, on average, higher in sole soy than sole wheat (p = 0.025). Soil pH was significantly higher in the (unplanted) sole soy than intercropping (*p* = 0.047) and sole wheat (p < 0.001) at Sampling 1 and 2 (*p* < 0.001 for intercropping and sole wheat) as soy was not planted until sampling 3. Intercropping had a significantly lower soil pH than sole wheat at sampling 2 (p = 0.01) and sole soy at sampling 4 (p = 0.005). The net effect ratio (NER) for pH remained at nearly 1 for every sampling period (Table 1).

**Table 1:** Average net effect ratio (NER) values for each sampling based on the difference in values between the intercropping and sole cropping treatments.

Indicator	Sampling 1	Sampling 2	Sampling 3	Sampling 4	Sampling 5
pH	1.01 (0.00)	0.98 (0.00)	1.02 (0.02)	1.02 (0.01)	1.01 (0.03)
POXC^1^	1.07 (0.08)	1.02 (0.07)	0.97 (0.08)	1.04 (0.04)	1.00 (0.05)
16S copy number	0.89 (0.10)	1.08 (0.06)	1.07 (0.09)	1.19 (0.24)	1.13 (0.20)
ITS2 copy number	1.42 (0.26)	1.32 (0.33)	0.66 (0.14)	1.41 (0.34)	1.03 (0.18)
AWCD^2^	0.82 (0.09)	1.31 (0.12; t = 2.59, p = 0.049)	1.02 (0.10)	1.14 (0.23)	1.42 (0.60)
Infiltration					1.14 (0.50)

Standard errors, and p values if the indicator had a significant value at the sampling point, are in parentheses.

^1^Permanganate-oxidizable carbon.

^2^Average well color development.

We found no difference in POXC between treatments. However, we did find an effect of sampling time on POXC (p = 0.005) with POXC at Sampling 2 (p = 0.01, 316 mgC kg soil^−1^) and Sampling 3 (p = 0.003, 312 mgC kg soil^−1^) significantly lower than Sampling 4 (353 mgC kg soil^−1^; Fig. 2). The NER for POXC remained was between 0.97 and 1.06 throughout the year and was not significantly different than 1 (Table 1). POXC at sampling 5 was positively, significantly correlated with MAOM C (Pearson’s *r* = 0.69, p = 0.0001; Supplementary Fig. 3), MAOM N (Pearson’s *r* = 0.63, p = 0.005), and pH (Pearson’s *r* = 0.57, p = 0.013).

### Soil health physical indicator

**Figure 3:**
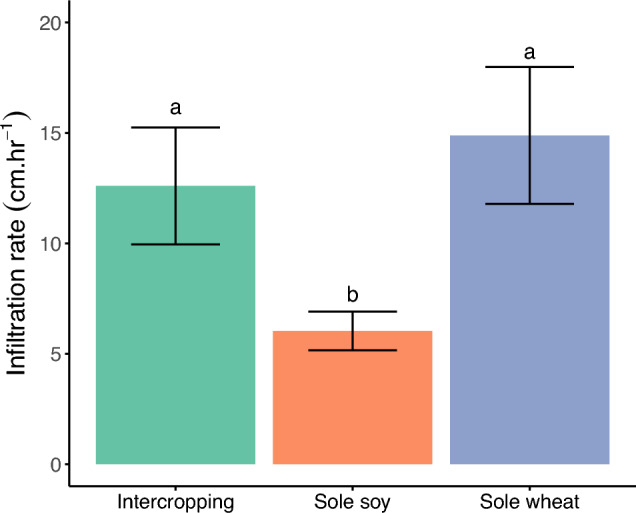
Rates of near-saturated soil water infiltration in each cropping system at Sampling 5.

There were significant differences in saturated soil water infiltration rates between treatments (*p* = 0.017; Fig. 3) with sole soy having a significantly lower infiltration rate than sole wheat (p = 0.004) and intercropping (p = 0.034). Sole soy’s average infiltration rate was 71% lower than intercropping and 85% lower than sole wheat while the percent difference between sole wheat and intercropping was only 16%.

### Soil health biological indicators

**Figure 4:**
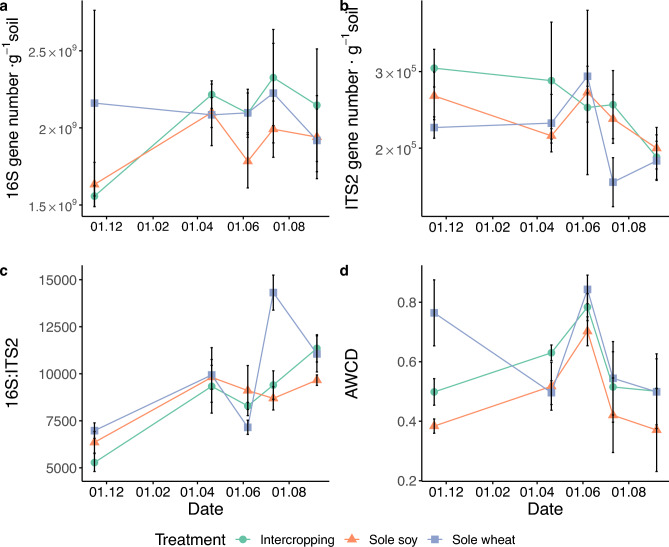
(**A**) Bacterial 16S rRNA gene number, (**B**) fungal ITS2 gene number, (**C**) 16S:ITS2 gene number ratio, and (**D**) average well color development (AWCD) of ecoplates over time. Bars show standard error.

Bacterial abundance based on 16S rRNA gene number was significantly higher in Sampling 2 (*p* = 0.044) and 4 (*p* = 0.028) compared to Sampling 1 (Fig. 4) but we found no significant differences between treatments. Fungal abundance was significantly lower at Sampling 5 compared to the initial sampling (*p* = 0.005; Fig. 4). The Bacteria: Fungi abundance ratio, which was calculated by the ratio of 16S:ITS2 gene copy numbers, was significantly affected by sampling time (p < 0.001; Fig. 4) with Sampling 1 lower than every other time (p < 0.01 for all pairwise comparisons). The Bacteria:Fungi abundance ratio was higher in sole wheat than intercropping (p < 0.0001) and sole soy (p < 0.0001) at Sampling 4 while intercropping and sole soy were not different (p = 0.79). The difference at the end of the wheat cropping system (Sampling 4) was driven primarily by higher ITS2 gene numbers in intercropping (intercropping average—2.57 × 10^5^, sole wheat average—2.09 × 10^5^ gene copies per gram soil) as there was less of a difference in 16S rRNA gene number (Intercropping average—2.33 × 10^9^, sole wheat average—2.23 × 10^9^ gene numbers per gram soil) between systems (Fig. 4). The 16S rRNA gene copy number at soil sampling 5 was negatively correlated with POMC (Pearson’s *r* = − 0.50, p = 0.01) and POMN (Pearson’s *r* = − 0.53, p = 0.023) as was the ITS2 gene copy number with POMC (Pearson’s *r* = − 0.67, p = 0.002) and POM N (Pearson’s *r* = − 0.61, p = 0.007).

Functional diversity of microbial communities proxied by EcoPlates substrate usage varied from 1 to 25 (of a maximum of 31) but there was no effect of treatment. There was a significant effect of sampling time (p < 0.0001) on average well color development (AWCD). AWCD was significantly higher at sampling 3 than the initial sampling (p = 0.04), sampling 4 (p = 0.002) and sampling 5 (p = 0.0003). Sole wheat had a higher AWCD than the unplanted soy plots at sampling 1 (p = 0.009). We found no significant effects of treatment on the different carbon substrate groups (amino acids, amines & amides, carbohydrates, carboxylic & acetic acids, polymers) as there was strong variation in group AWCD response (Supplementary Table 3). The NER for AWCD fluctuated with the initial value of 0.82 and reaching its highest value at Sampling 5 (1.42) indicating a higher AWCD for intercropping plots compared to the expected value from sole crops, but this was only significant at Sampling 2. (Table 1).

### Agronomic performance

The intercropped winter wheat over-yielded in terms of biomass and yield as it produced 80% of the sole cropped wheat biomass and 63% of the grain yield despite its 50% reduction in plant density compared to sole wheat plots. Nevertheless, the intercropped soy under-yielded due to slow growth and low biomass compared to the sole soy (Table 2). The land equivalent ratio (LER) based on crop biomass was 0.93 while the transgressive overyielding index (TOI) was 0.89 and the NER 1.12. When based on grain yield, the LER was 0.88, the TOI 0.73, and the NER 1.12.

**Table 2:** Total plant biomass and grain yield per system; standard errors are given in parentheses.

System	Wheat biomass (kg ha^−1^)	Soy biomass (kg ha^−1^)	Wheat yield (t ha^−1^)	Soy yield (t ha^−1^)
Sole cropping	10,871 (661)	7180 (931)	5.88 (0.24)	2.31 (0.23)
Intercropping	8713 (225)	944 (219)	3.74 (0.58)	0.56 (0.42)

## Discussion

### Chemical indicators for assessing soil health

We used relative changes in soil carbon fractions from the start to the end of the growing season as a sensitive indicator for short-term effects on soil health. Fractionation revealed that intercropping and sole soy increased MAOM C:N ratios compared to sole wheat as MAOM C increased, on average, in intercropping and sole soy while sole wheat decreased MAOM C. The change in MAOM C was significantly higher in sole soy than sole wheat, suggesting that including soy in the cropping system may be more advantageous for soil C storage compared to wheat but longer term studies are needed to see if this result persists as this is a single year study. MAOM is generally believed to originate from microbial sources and root exudates^32^ and higher quality substrates (lower C:N ratio) have been shown to increase MAOM C^33^ suggesting that the addition of nitrogen-rich crop biomass and nitrogen-supporting exudates from soy might have contributed to the development of MAOM C. The addition of legumes to a continuous grain cropping system in the American Midwest was found to increase MAOM C which was partially attributed to legume exudates and lower C:N ratio of legume residue^34^. Given that MAOM is considered a more stable C pool^35^, the modest, short-term increases in MAOM C and MAOM C:N ratios suggest that C fractionation can be a sensitive soil health indicator as other studies have found changes in MAOM C levels after 6 months—2 years^36–38^ with the short-term increases in MAOM C still persisting after 5 years^37^.

In contrast, we observed no differences in POM between any of the cropping systems. Numerous studies have demonstrated that intercropping enhances overall SOC^5,15,39,40^; however, investigations specifically on POM dynamics in other intercropping systems have yielded inconclusive results^39,41^. POM C is thought to originate from plants, and since the biomass inputs of the intercropping system were nearly equivalent to those of sole cropping (LER = 0.93), perhaps the differences in input biomass were insufficient to impact our soils. Our soils are extremely sandy (60–70% sand) with < 1% SOC and soils low in SOC tend to be dominated by MAOM rather than POM^42^. Nevertheless, POM C and N fractions did, in general, increase amongst all treatments over the growing season indicating that the presence of any living crop on the field was beneficial for POM accumulation.

Similarly to POM, POXC levels in our soils were low and unaffected by cropping system. We saw only a slight temporal pattern with low level of POXC in June and a peak in July. In a study looking at different cropping systems from May to October, Culman et al.^43^ also found a similar late summer peak in POXC. Slight positive relationships between crop diversity and POXC have been found^44,45^ but other studies found no effect of crop rotation or intercropping on POXC, instead POXC was related to total SOC levels^46^. We found a positive correlation between POXC and MAOM C fractions suggesting a relationship between C pools, although there was no relationship to POM C. Nevertheless, POXC can be difficult to measure in soils with low SOC^47^ and our soils may be too low in SOC to see any noticeable difference of management in a single season. We found significant treatment differences on pH at the earlier sampling dates, which likely was due to fertilization of wheat. Fertilization with ammonium and urea fertilizer has been shown to reduce soil pH^48,49^ and this aligns with the pH drop in our trial as significant changes in pH only occurred at sampling 2, approximately when fertilizer was applied.

### Soil water infiltration as a physical indicator for assessing soil health

While intercropping was similar to soy in terms of C fractions, intercropping’s soil water infiltration was more similar to sole wheat. Infiltration in the intercropping plots was only 16% lower than sole wheat but 71% higher than sole soy despite the 50% reduction in wheat density in relay intercropping. In a study looking at root distributions of different crops, the R50 (depth where 50% of a plant’s roots reached) was 42% deeper in wheat than soy^50^, so wheat rooting patterns may have contributed to the infiltration results. Managing soil water is imperative in our sandy soils as summer storms are common, leading to 20–50 mm water per day or per hour in exceptional cases. Cropping systems that support higher infiltration can better support these rainfall events rather than leading to erosion. Crop rotational diversity effects on infiltration are inconsistent^51^ with more benefits from cover crops and practices that ensure continuous soil cover and living roots^51^—like relay intercropping can. Data on soil water dynamics in cereal-legume intercropping is extremely rare^52^. To our knowledge, our study is the first on wheat-soy relay intercropping and results indicate that soil water infiltration can be an indicator able to differentiate management effects relatively quickly.

### Biological indicators for assessing soil health

Bacterial rRNA 16S gene abundance peaked in June whereas fungal ITS2 gene abundance decreased over time, albeit with a small increase in June, the period for maximum growth for wheat. Mixed cropping can increase microbial biomass C (MBC)^41^ as the diverse root exudates can support microbial activity and abundance^17–19^. Intercropping soy with wheat in a pot experiment increased both microbial and fungal diversity compared to sole crops which was positively correlated to the higher root dry biomass found in the intercropping treatment^21^. Nevertheless, we did not find significant treatment differences on microbial abundance. Audu et al.^39^ found no difference in MBC and lower 16S rRNA gene abundance in intercropping but did find a significant relationship between 16S rRNA gene abundance and POM C, indicating that microbial biomass can be a function of available C. However, we found a moderate negative relationship between microbial gene abundance and POM fractions. The addition of switchgrass into pine plantations led to a decrease in POM C but an increase in microbial biomass, suggesting that switchgrass brought about POM decomposition through a priming effect of the soil microbial community^53^ and a similar priming effect could have occurred in our study site. Nevertheless the differing relationships between POM C and microbial abundance could also be affected by factors that were different between the studies including including soil type, crop rotational history, and soil sampling time.

Increasing microbial biomass in soils often implies healthier soils; however, the composition of the microbial community matters. Conventional agricultural practices such as intensive tillage and synthetic fertilizers have also shown to increase bacterial abundance and decrease fungal abundance^54,55^. Fungi are more sensitive to disturbance but play key roles in soil processes such as decomposition and C storage. At the time of our wheat harvest in July, sole wheat had the highest Bacteria: Fungi ratio. The lower Bacteria: Fungi ratio in intercropping and sole soy was driven by higher fungal abundance in intercropping as the treatment also had a high average bacterial abundance, while sole soy had high levels of fungal abundance but the lowest average bacterial abundance. The still-growing soy in intercropping and sole soy plots after wheat harvest possibly supported fungi through N-rich exudates and the fact that there was still actively growing soil cover suggesting that fungal activity is more reliant on the presence of living roots, especially in our sandy soils. As soy and wheat are functionally very different plants, we would expect a difference in microbial communities but microbial functional diversity and activity did not vary between treatments. Ecoplate functional diversity may be too coarse of a method considering it only screens culturable, aerobic microorganisms or the bulk soil too coarse of a sampling. Finer methods, such as measurements on rhizosphere soil, might be more appropriate for single season studies.

### Agronomic performance of soy-wheat cropping systems

The TOI of the system was 0.89 indicating that the intercropping system produced nearly 90% of the biomass as the most productive crop, wheat while the LER of 0.93 shows that our intercropped system did not over yield compared to the sole crops. Intercropping systems tend to over yield compared to sole crop systems with a LER up to 1.29 ± 0.02 in meta-analyses^56,57^, which is one of their primary benefits. Our system struggled due to climatic conditions affecting the intercropped soy. The intercropped wheat over yielded but the intercropped soy plant growth was stunted. Intercropping yields can be highly influenced by resource competition of light, water, and nutrients as crops can compete for the same resources^25^. The poor performance of intercropped soy was likely due to high heat and drought in May and June as drought during soy establishment is shown to be very important for soy success^58^ and soil moisture levels were similar between treatments later in the season.

### Sensitivity of indicators for temporal soil health dynamics

Our results show temporal patterns of soil health indicators, highlighting the dynamic nature of these indicators. Other studies tracking soil indicators throughout a growing season found similar peaks of biologically active nutrient pools in the later summer^43,59^ but such studies are uncommon, more so in intercropping literature. Although this method might require more time, it is valuable for systems with multiple crops with distinct management and phenologies. The choice of sampling would influence whether differences are found and what potential mechanisms could be. For instance, differences between sole soy and intercropping during Sampling 1 likely stem from the presence or absence of roots, whereas later samplings may reflect more on crop diversity. Exploring longer-term experiments integrated into a realistic crop rotation will provide a more comprehensive understanding of our relay intercropping system but our soil sampling throughout the year long period offers initial insight into the dynamic patterns of these indicators. Delving deeper into mechanisms behind soil health indicators by measuring, for example, root biomass allocations or root exudates could elucidate the underlying processes behind intercropping’s success and shortcomings.

## Conclusion

This paper is the first study on soy-relay intercropping’s impacts on soil health and an investigation into appropriate indicators for monitoring short-term management changes. Notably, the intercropping system did not exhibit any adverse effects and managed to maintain several favorable soil characteristics of both sole soy and sole wheat systems. Given that some of the soil properties we measured are generally slower to change, these short-term changes are early signals in the direction of change and suggest that they are useful indicators for our soils. In addition, the temporal aspect of our study can also serve as a useful framework for other studies of relay intercropping that, in general, could provide more benefits to soil health if designed well and with conditions (i.e. more rainfall) conducive to its success. Despite the negative effects of heat and drought on intercropped soy, soy-wheat relay intercropping may have potential benefits to soil health compared to sole crops that warrant additional studies.

## Methods

### Field experiment

Soy-wheat relay intercropping trials were conducted at the Experimental Field Station of the Leibniz Center for Agricultural Landscape Research (ZALF), Müncheberg, Germany, 50 km east of Berlin during the 2021–2022 cropping season. All procedures were conducted in accordance with local guidelines and no permissions were needed to collect plant samples. Soils in the region are sandy loams formed from glacial deposits. The soil type is classified as Haplic Albeluvisol^60^ with an average of 64% sand, 8% clay, 28% silt and 0.51% total carbon. The long-term average annual temperature is 9.0 °C with an average annual precipitation of 563 mm (Supplementary Fig. 1).

**Figure 5:**
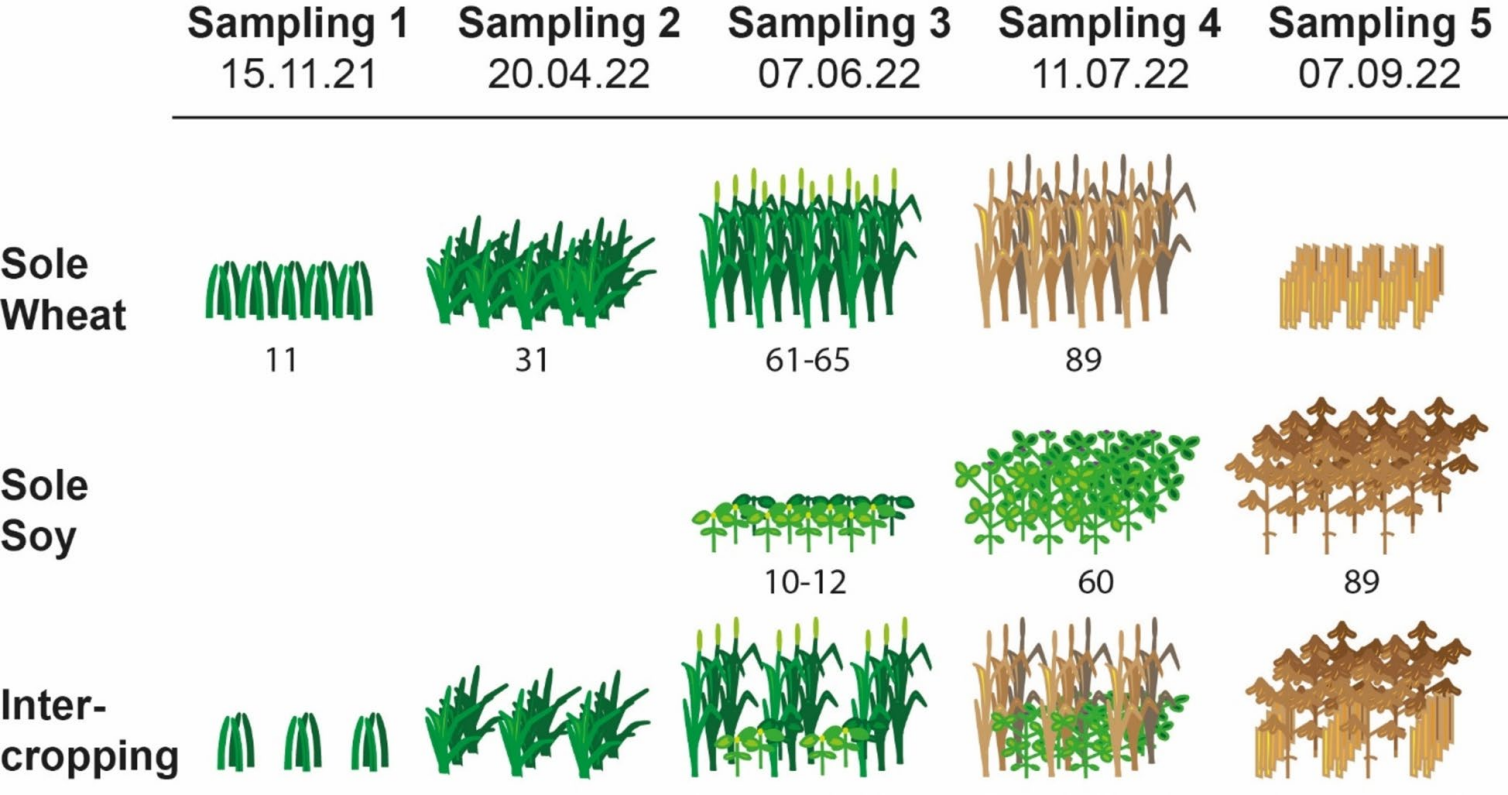
Schematic of sole wheat, relay intercropping, and sole soy plots during the year-long soil sampling. Numbers refer to BBCH.

Our experiment consisted of a randomized, blocked plot trial with 3 cropping treatments and 6 replicates per treatment spread over 6 blocks. Plots of 3 × 8 m were sown with sole winter wheat *cv.* Moschus, sole soy *cv.* Merlin, or soy-wheat relay intercropping. Seeds were obtained from a local commercial source (Agravis). A 50 cm buffer strip of wheat was left between all plots with the exception of sole soy plots which had additional adjacent 3 × 8 m plots as buffers in order to allow crop specific management (e.g. fertilizer application) and to minimize effects (e.g. from nitrogen fixation) on neighboring plots (Supplementary Fig. 2). Wheat was planted in 12.5 cm rows, 2 cm deep at 416 seeds m^2^. For soy-wheat relay intercropping, the winter wheat was planted in alternating 12.5 cm double rows with a 37.5 cm gap for soy to be drilled into in the spring (Fig. 5). Sole soy was sown in 50 cm rows with a density of 70 plants m^2^ at 3–4 cm depth. Intercropped soy was seeded with a density of 70 plants m^2^ and a shallow sowing depth of around 2 cm when winter wheat was in the tillering stage and before stem elongation to avoid damage by the tractor wheels. All crops were managed conventionally with mineral fertilization for wheat and herbicides for both crops (Supplementary Table 1). Plots received irrigation in the late spring and summer following soy irrigation schedules with overhead irrigation. At the end of the growing season prior to crop harvest, we collected a 0.5 m^2^ quadrat from each plot to estimate total above-ground biomass per plot. Grain was harvested with a combine harvester. Wheat straw was left on the field until the end of the trial in both intercropping and sole wheat plots.

### Soil sampling

Soil health indicators were measured along an entire relay intercropping season shortly after the winter wheat sowing in November 2021 to soybean maturation in September 2022. Soil was sampled at five key points in the crops' developmental periods, at each sampling for all treatments for a total of 90 samples (Fig. 2). The sampling points included the approximate sowing and harvest times for each crop, as well as points of rapid growth (e.g. Sampling 2 wheat stem elongation). At each time point, soil was collected with an auger from 15 equi-distance points per plot from 0–15 cm depth and homogenized to make one composite sample per plot. Samples were always taken between rows of plants, for intercropping this was between a row of soy and wheat. Care was taken to always take samples from precisely in the middle of rows to not bias results towards soy or wheat. Soil was kept cool until returned to the lab and subsamples were immediately frozen at − 80 °C for further downstream analysis. Another subset of fresh soil was analyzed for community level physiological profiling. The remainder of the sample was left to air dry at room temperature.

### Soil analysis

We selected 7 soil health indicators spanning chemical, physical, and biological soil properties that are known to be sensitive to management, reasonably affordable, and informative to soil ecosystem services of interest^13,61^ (Table 3) in sandy soils and low precipitation regions (i.e. water regulation and carbon storage).

**Table 3:** Soil health indicators used in the study and their relevance to soil and crop health.

Indicator	Relevance to soil	Relevance to plants
pH	Controls nutrient availability, influences microbial communities, and soil processes	A soil pH of 5.5–7 is ideal for most crops to obtain necessary nutrients
POXC	Measure of reactive carbon pool readily accessible to microbes and is very strongly correlated with other indicators of soil health like total organic C (TOC)^62^	Soil carbon pools are important for maintaining soil function imperative to plant growth, shown to affect agronomic performance^63^
Carbon fractions	Changes in soil organic carbon (SOC) require decades. C fractionation can show faster changes (Particulate organic matter- POM) as well as indicate the stability of the soil C (mineral-associated organic matter- MAOM). POM also plays roles in soil aggregation and infiltration	POM is primary source of plant available N. SOC is important for controlling soil functions (e.g. water and nutrient regulation) imperative for plant growth
Water infiltration	The amount of water able to move though soil and is related to soil compaction, pore space, and water retention abilities	Important component of plant water availability and compaction affecting root growth
Microbial abundance	Microbes are the base of the trophic chain and important players in decomposition, nutrient availability, and C storage	Plants benefit from easily available nutrients, increased SOC, and improved soil structure supported by microbial processes. Increased microbial diversity has been linked to plant health and growth^64^
Microbial diversity	Higher microbial functional diversity can support more soil processes and healthier soils^65^	

Soil pH was measured in water with 10 g of air-dried soil. POXC was measured with 2.5 g of air-dried, sieved soil with 20 ml 0.2 M KMnO_4_ for 2 min then left to settle in the dark for 10 min. Then 1 ml of supernatant was diluted with 49 ml of deionized water and the dilution absorbance measured at 550 nm.

Air dried and sieved soil was fractionated by size to obtain POM and MAOM carbon fractions according to the procedure by Cotrufo et al.^42^. We shook 15 g of soil with glass beads in 90 ml 0.5% (NaPO_3_)_6_ for 18 h. Soil was fractionated with a 53 µm sieve with deionized water. POM is defined as the organic soil fraction > 53 µm and MAOM as organic soil fraction ≤ 53 µm. Fractionated soils were ground and analyzed for total C and N with an elemental analyzer (Leco Instruments GmbH). Due to the slower dynamics of C accumulation, only the sampling time points 1, 4, and 5 were fractionated to compare the effects between the initial soil state (sampling 1 for all crops) and the effects of an entire cropping season (up to sampling 4 for sole wheat and 5 for intercropping and sole soy).

We measured soil water infiltration with a hood infiltrometer (Umwelt-Geräte-Technik)^66^. Infiltration measurements were taken at sampling time 5 as the soil was too dry for accurate measurements earlier in the season. Due to the time intensive protocol of the hood infiltrometer, one measurement was taken per plot over a 3 day period. The 12.4 cm radius hood was placed between rows of crops in the center of the plot; any vegetation in the area was cut to ground level. The hood’s base ring was pushed 5 mm into the soil. Water infiltrated through the system at ambient pressure until the infiltration rate equalized. Once the soil was saturated and the readings steady, 20 measurements were taken to calculate the infiltration rate.

We measured fungal and bacterial abundance in soil with qPCR which has been shown to accurately track overall microbial abundance^67,68^. DNA was extracted from frozen soil samples with a DNEasy PowerLyzer PowerSoil Kit (Qiagen) according to manufacturer instructions. DNA sample quality was checked with a NanoDrop (ThermoFisher Scientific) before qPCR. 2 µg of template DNA was added to 10 µl Luna qPCR master mix (New England Biolabs), 7 µl sterile DNA-free H2O and 0.5 µl of each 100 pmol uµl^−1^ forward and reverse primer. We used the 16 s V4 primers 799F and 1115R to measure bacterial abundance and the ITS2 primers ITS86F and ITS4R for fungal abundance^69^. All reactions were carried out in duplicates on a qTower3 (Analytik Jena). The 16 s reaction was carried out under the following thermocycler conditions: 95 °C for 2 min, 40 cycles of 95 °C for 15 s, 54 °C for 30 s, and 72 °C for 1 min followed by a final 5 min at 72 °C. The ITS thermocycler conditions were: 95 °C for 2 min, 40 cycles of 95 °C for 30 s, 55 °C for 30 s, and 72 °C for 40 s followed by a final 5 min at 72 °C.

We utilized Ecoplates (Biolog, USA) to estimate microbial community-level physiological profiling (CLPP) of soil, a measure of functional diversity. Ecoplates contain 31 different carbon substrates from 5 substrate categories (amines & amides, amino acids, carbohydrates, carboxylic & acetic acids, and polymers). Briefly, 5 g of fresh soil was mixed with 45 ml of sterile 0.9% NaCl and 150 µl of the supernatant was used to inoculate each well of the Ecoplate. Color development of the plates, from tetrazolium violet redox dye in the wells, was measured every 24 h for 7 days at 590 nm absorbance. Color development at day 4 was selected for subsequent analyses as it showed the maximum dye utilization. Wells with absorbance > 0.25 were counted as a positive value^70^. Average well color development (AWCD) was also calculated to measure carbon substrate usage patterns between samples according to:

$$AWCD= \sum \frac{{c}_{i}}{n}$$

where *C* is the color development of an individual well and *n* is the number of substrates. AWCD represents the overall diversity of substrate use of a microbial community with a higher value indicating more substrates were used and a low value indicating few substrates were used. AWCD for each of the five carbon groups were calculated in the same way.

### Statistical analyses

Treatment differences between soil health indicators measured once (soil water infiltration, change in carbon fractions) were analyzed with mixed effect models with the *lmer* function from the *lmerTest* package in R (v.4.2.2, R Core Team). Cropping treatment was set as a fixed factor and experimental block as a random factor to account for local differences in soil texture. For indicators measured several times (pH, POXC, ecoplate activity), mixed effect models were also employed but with sampling time as an additional fixed factor and plot as a random factor to act as a repeated measure as samples were taken from the same plot over time. Residuals versus fitted values and normal quantile–quantile (QQ) plots were used as model diagnostics to assess normality of residuals and homogeneity of variance. Data was log or square-root transformed when assumptions were not met. When we found significant effects of sampling time and treatment on soil health indicators measured multiple times, we then utilized mixed effect models on data from each sampling time point individually to analyze treatment differences at each sampling time. We analyzed indicators with count data (microbial abundance and ecoplate functional richness) with generalized linear models with a negative-binomial distribution with the R package *lme4.* Cropping system and sampling time were fixed factors with block as a random factor and plot as a random, repeated factor. Tukey HSD post-hoc tests were used to determine variable level differences on statistically significant variables (p < 0.05) with the *multcomp* package. Pearson correlation coefficients between indicators and yield at Sampling 5 were calculated to see relationships between soil health indicators. Correlations were only performed on Sampling 5 data as this was the time point with all indicator measurements and it represented the values after an entire season.

We calculated the net effect ratio (NER) which is the ratio of the observed intercropping treatment to the expected value based off of the sole crops weighted by their respective proportion. We used the following formula:

$$NER=\frac{{V}_{IC}}{{P}_{S}\cdot {V}_{S}+{{P}_{w}\cdot V}_{W}}$$

where *P*_*s*_ and *P*_*w*_ are the proportion of land for soy and wheat, respectively, in intercropping plots and V_S_ and V_W_ are the monoculture values of each indicator and V_IC_ is the observed value in the intercropping plot. A value was calculated for each block. A one-sample t-test was used to determine if the values were different from 1. A NER > 1 indicates higher than expected values for the intercropping plots compared to sole cropping. Land equivalent ratios (LER) for crop biomass and yield were calculated for each treatment according to the following formula, where IS and IW are intercropped soy and wheat and SS and SW are sole soy and wheat, respectively.

$$LER=\frac{{V}_{IS}}{{V}_{SS}}+\frac{{V}_{IW}}{{V}_{SW}}$$

The LER measures the relative amount of land needed for sole crops to produce the same total yield as intercropping per unit area^7^. The transgressive overyielding index (TOI) was calculated as a way to estimate the relative biomass and grain yield of the intercropping system compared to the most productive sole crop. TOI was calculated based on the following formula^7^ :

$$TOI=\frac{({V}_{IS}+{V}_{Iw})}{max({V}_{SW }, {V}_{Ss })}$$

## Supplementary Information

Supplementary Information.

## Data Availability

Data will be provided upon request. To obtain data please contact the corresponding author Jennifer B. Thompson at jennifer.thompson@zalf.de.
