# Supplementary material for: Seasonal soil health dynamics in soy-wheat relay intercropping
Source: Sci Rep. 2024 Aug 16;14:18989. doi: 10.1038/s41598-024-69903-5 (PMC11333471; doi:10.1038/s41598-024-69903-5)
Supplement: Supplementary file 1 — Supplementary Information. [file 41598_2024_69903_MOESM1_ESM.pdf]

Supplemental materials for: **Seasonal soil health dynamics in soy-wheat relay intercropping**

Jennifer B. Thompson, Thomas F. Döring, Timothy M. Bowles, Steffen Kolb, Sonoko Bellingrath-Kimura, Moritz Reckling

Corresponding author: Jennifer B. Thompson, jennifer.thompson@zalf.de

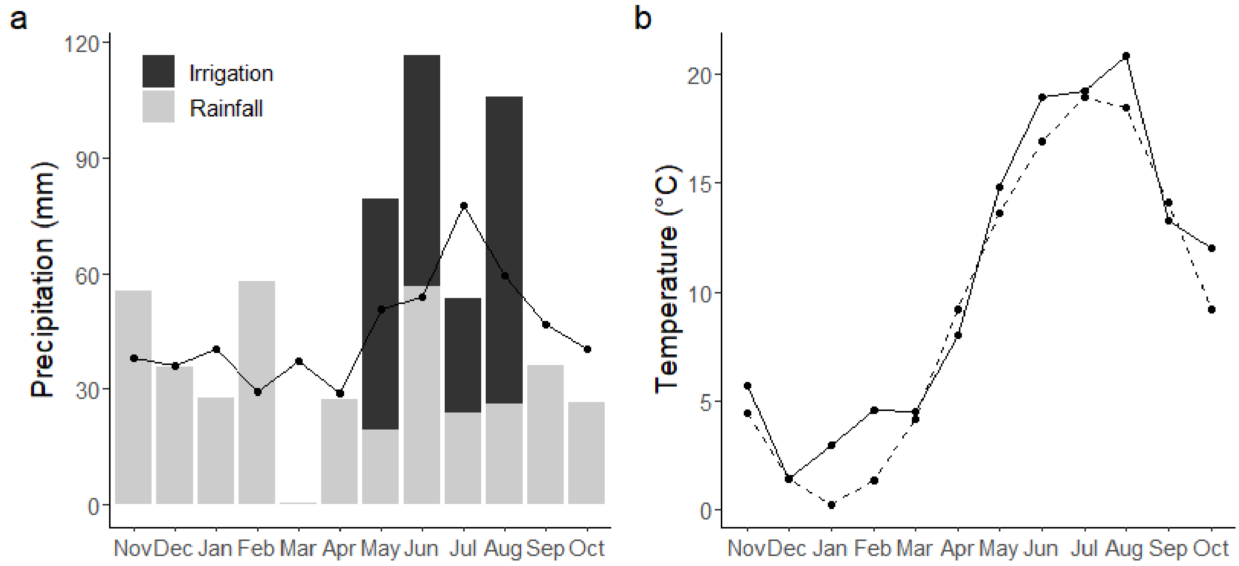

Figure S1 a) Precipitation and irrigation from November 2021-October 2022. The black line represents the historical rainfall average from 1990-2022, b) Average monthly temperatures from the 2021-2022 growing season (solid line) and the historical temperature average from 1990-2022 (dashed line).

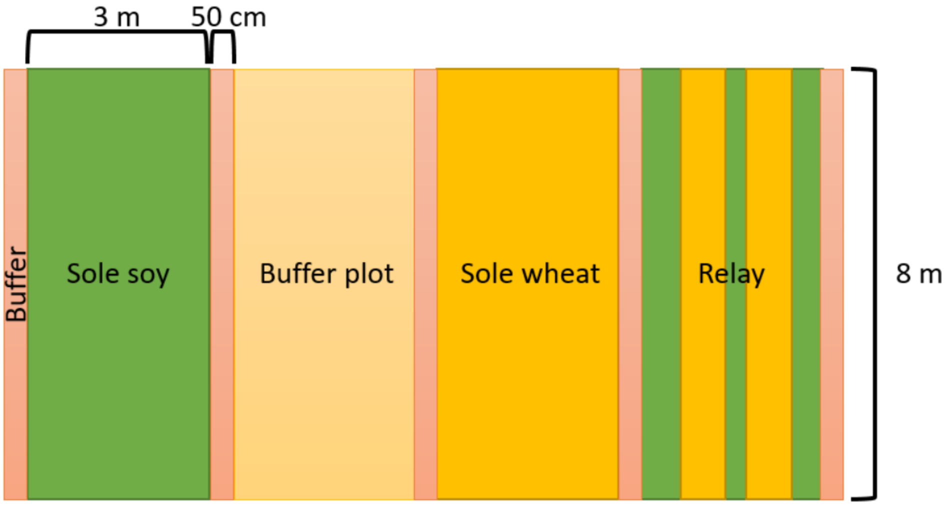

Figure S2. Example of the plot and buffer layout in one experimental block of the study. Blocks were replicated six times with plots randomly ordered throughout. Both the buffer and buffer plots were cultivated with winter wheat but received no management (i.e. fertilization, herbicides, etc).

Table S1 Management of the 3 cropping systems.

**Practices**	**Sole wheat**	**Soy-wheat relay intercropping**	**Sole soy**
Tillage	Previous crop rye. All plots plowed at a 23 cm soil depth with a mouldboard plough in early October		
Seedbed preparation	Leveled to 10cm with a cultivator		Cultivated to 5 cm
Sowing	14.10.2021<br>Cereal drill	Same as sole crops<br>Cereal drill and direct drill	29.04.22<br>Direct drill
Fertilization	60 kg/ha N, 14 kg/ha S in March. 40 kg/ha N, 10 kg/ha S in April<br><br>(N is a mix of urea, NH_4^+, and NO_3)	60 kg/ha N and 14 kg/ha S in March<br><br>*N fertilization was reduced compared to sole wheat for adjusted wheat density to avoid N losses	No fertilization
Herbicides	Carmina 640 (1.50 l/ha) was applied at BBCH 11 in a broth of 600 l/ha. The growth regulator Acucel (1.00 l/ha) was applied on the same plots in a 400 l/ha broth at BBCH 30		Pre-emergence herbicides (Centium 36 CS 0.25 l/ha, Sencor Liquid 0.30 l/ha and Spectrum 0.60 l/ha) applied BBCH 03 in a 600 l/ha broth. Post emergence herbicide (Fusilade MAX, 2.00 l/ha) applied in 600 l/ha broth.
Harvest	21.07.2022	Wheat: 21.07.22<br>Harvested with a FlexxiSelect combine header attachment (Flexxifinger).<br>Soy: 08.11.22	12.10.2022

Table S2 Absolute values of carbon fractionation % C and %N from the final sampling point of each cropping system.

Treatment	MAOM %C	MAOM %N	MAOM C:N	POM %C	POM %N	POM C:N
Sole wheat	1.72	0.18	10.1	0.28	0.02	11.2
Intercropping	1.85	0.18	10.4	0.32	0.03	10.8
Sole soy	2.02	0.18	10.7	0.29	0.03	10.7

Table S3 Substrate group average well color development (AWCD) from ecoplates

Sampling time	Treatment	Amines & Amides	Amino acids	Carbo-hydrates	Carboxylic & Acetic acids	Polymers
1	Intercropping	0.536	0.429	0.380	0.567	0.401
	Sole soy	0.314	0.298	0.278	0.456	0.428
	Sole wheat	0.370	0.514	0.897	0.788	0.565
2	Intercropping	0.666	0.520	0.390	0.808	0.587
	Sole soy	0.368	0.3449	0.468	0.601	0.528
	Sole wheat	0.557	0.376	0.361	0.627	0.408
3	Intercropping	0.761	0.596	0.675	0.834	0.789
	Sole soy	0.570	0.612	0.529	0.804	0.648
	Sole wheat	0.637	0.602	0.741	1.003	0.752
4	Intercropping	0.602	0.402	0.567	0.428	0.407
	Sole soy	0.525	0.278	0.496	0.346	0.350
	Sole wheat	0.470	0.372	0.579	0.523	0.521
5	Intercropping	0.362	0.278	0.566	0.568	0.381
	Sole soy	0.475	0.252	0.433	0.284	0.348
	Sole wheat	0.357	0.323	0.558	0.515	0.405

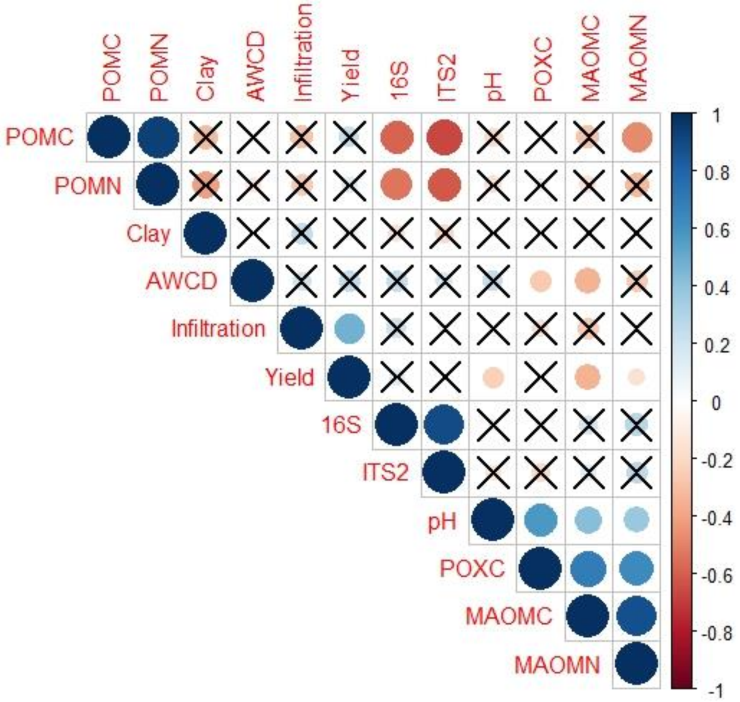

Figure S3 Pearson's correlations of soil health indicators and yield from Sampling 5. Non-significant relationships are crossed with an 'x' and only significant moderate (r>0.35) relationships are mentioned in the main text.
